# Novel splice variants associated with one of the zebrafish *dnmt3 *genes

**DOI:** 10.1186/1471-213X-5-23

**Published:** 2005-10-19

**Authors:** Tamara HL Smith, Christine C Dueck, Aizeddin A Mhanni, Ross A McGowan

**Affiliations:** 1 Dept. of Biochemistry, Memorial University, St. John's, NL, Canada; 2 Faculty of Medicine, University of Manitoba, Winnipeg, MB., Canada; 3 Department of Pediatrics & Child Health and Biochemistry and Medical Genetics, University of Manitoba, Winnipeg, MB., Canada

## Abstract

**Background:**

DNA methylation and the methyltransferases are known to be important in vertebrate development and this may be particularly true for the Dnmt3 family of enzymes because they are thought to be the *de novo *methyltransferases. Mammals have three Dnmt3 genes; Dnmt3a, Dnmt3b, and Dnmt3L, two of which encode active enzymes and one of which produces an inactive but necessary cofactor. However, due to multiple promoter use and alternative splicing there are actually a number of dnmt3 isoforms present. Six different dnmt3 genes have recently been identified in zebrafish.

**Results:**

We have examined two of the dnmt3 genes in zebrafish that are located in close proximity in the same linkage group and we find that the two genes are more similar to each other than they are to the other zebrafish dnmt3 genes. We have found evidence for the existence of several different splice variants and alternative splice sites associated with one of the two genes and have examined the relative expression of these genes/variants in a number of zebrafish developmental stages and tissues.

**Conclusion:**

The similarity of the dnmt3-1 and dnmt3-2 genes suggests that they arose due to a relatively recent gene duplication event. The presence of alternative splice and start sites, reminiscent of what is seen with the human DNMT3s, demonstrates strong parallels between the control/function of these genes across vertebrate species. The dynamic expression levels of these genes/variants suggest that they may well play a role in early development and this is particularly true for dnmt3-2-1 and dnmt3-1. dnmt3-2-1 is the predominantly expressed form prior to zygotic gene activation whereas dnmt3-1 predominates post zygotic gene activation suggesting a distinct developmental role for each.

## Background

The epigenetic modification of DNA by the addition of a methyl group to the 5 position of cytosine is an important mechanism for control of gene expression in vertebrates. This is particularly true during development where DNA methylation is thought to have a role in genome imprinting [1,2], X inactivation [3] and lineage determination [4]. Methylation has been most intensely studied in mammals where the levels have been shown to be quite dynamic during early development, decreasing soon after fertilization and increasing again by the gastrula stage [5,6]. The importance of this de-methylation/re-methylation cycle to the developmental process has been clearly demonstrated by perturbations of that methylation that generally leads to embryonic lethality [7,8]. Given the importance of methylation in sustaining normal early developmental processes, the enzymes that add and maintain that methylation are of significant interest. The dnmt3 family of methyltransferases that are thought to be important in *de novo *methylation (that is the addition of methyl groups to previously unmethylated sequences) are of particular interest in this context. There are three members of this family in mammals; two have catalytic activity, Dnmt3a and Dnmt3b; and the third, Dnmt3L, is important as a cofactor, particularly for the methylation of imprinted loci [9]. Functionally, however, the dnmt3 family is not limited to just three products because both the *Dmnt3a *and *b *transcripts can be alternatively spliced to generate a number of different RNAs. *Dnmt3a *has two splice variants differing in the 5' region whereas *dnmt3b *has a number of possible splicing products [10]. These variations in the dnmt3 proteins may allow for a greater diversity in the function and/or targets of these enzymes.

Methylation in zebrafish has recently been the focus of a number of reports, and methylation has been found to be dynamic during its early development [11]. Also, as in mammals, blocking re-methylation in zebrafish results in abnormal development and death [12].

The zebrafish actually has at least twice the mammalian number of dnmt3 genes; six have been submitted to databases so far (GenBank numbers AB196914, AB196915, AB196916, AB196917, AB196918, AB196919) [19]. The significance of the increase in dnmt3 gene copy number in zebrafish is unknown.

We have isolated and analysed a number of the zebrafish dnmt3 gene sequences and have identified two dnmt3 sequences that are located very close together in a single linkage group. The very close proximity of the two sequences provides an interesting opportunity to examine how the expression of these genes is controlled since one copy has a very limited upstream promoter region relative to the other.

## Results and Discussion

**Figure 1:**
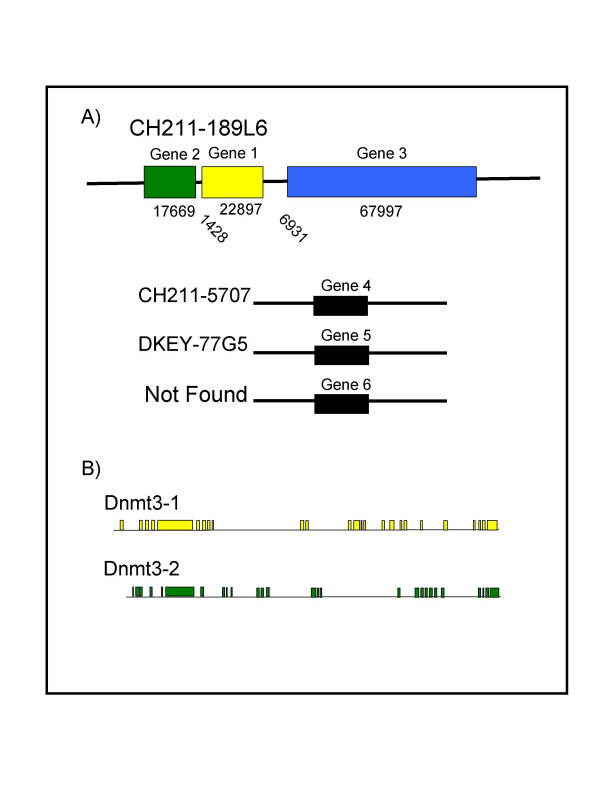
**The dnmt3 genes in zebrafish**. A) The Welcome Trust Sanger Institute library numbers are provided for all genes while the genomic size and distances are indicated for genes 1, 2, and 3. B) A more detailed view of the genomic structures of Gene 1 and Gene 2. Boxes represent exons and adjoining lines represent introns.

We used a dnmt3 sequence already present in the zebrafish EST database (GenBank number AF135438) to identify and isolate the complete cDNA sequences of four of the dnmt3 genes found in the zebrafish. Three of these are located in the same linkage group (linkage group 23) and two of them very closely juxtaposed to each other (Figure 1). The very close proximity of those two genes has some interesting implications with respect to their origin and the control of their expression, given the much more limited potential promoter region of one relative to the other. We, therefore, undertook a closer examination of the two genes, which we named dnmt3-1 and dnmt3-2.

**Figure 2:**
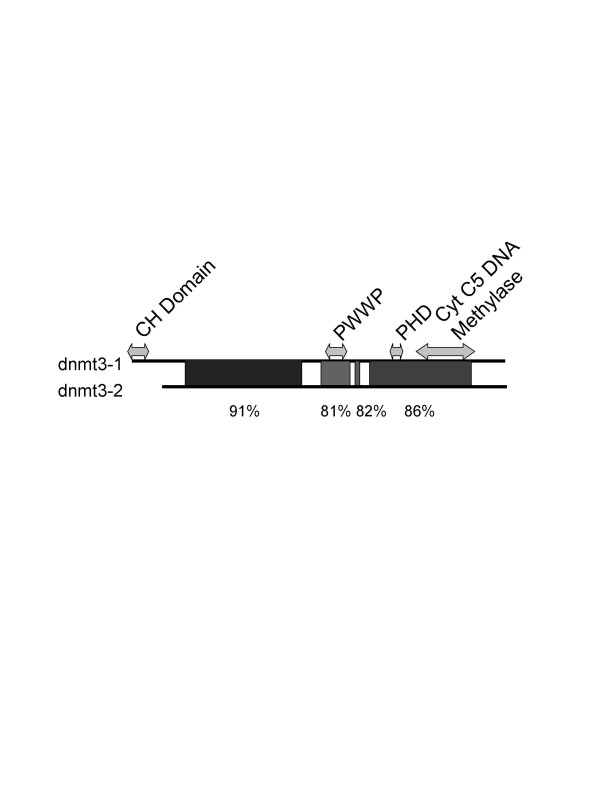
**Homology between Gene 1 and Gene 2 at the nucleotide level**. Percentages above 80% are shaded, and specific homology percentages are provided below the figure (numbers obtained by BLAST alignment, NCBI). Arrows span the nucleotides that give rise to the identified motifs labelled above the respective arrows. Note: All identified motifs are characteristic of the C-5 methyltransferases, with the exception of the CH domain.

From the end of the polyA addition site of gene 1 to the beginning of our cloned sequence for gene 2 (probably not actually beginning at the cellular transcriptional start site) consists of only 1428 base pairs. Since there is only a small amount of 5' sequence that is associated with the dnmt3-2 gene this limits the control of the expression of this gene to a small and easily manipulated region. Analysis of this region suggests that it is a TATA-less promoter with a number of potential transcription factor binding sites including AP1 and SP1 binding sites which have also been reported for mammalian Dnmt3s [13,14]. The sequence of the cloned genes, dnmt3-1 and dnmt3-2 revealed open reading frames that could encode polypeptides of 1447 and 1297 amino acids, respectively. Comparison of the sequences of these two genes to zebrafish genomic maps present in the Genbank database allows for an analysis of the genomic structure. That structure along with the relative position of the two genes is shown in figure 1. The two genes are very similar in sequence; 72% at the nucleotide level and 74% identical at the amino acid level, with large regions being more than 80% identical (figure 2). This is in contrast to only 19–28% similarity at the nucleotide level, and 36–46% amino acid similarity when compared to the other dnmt3 sequences present in the zebrafish genome. This trend is also true for the conserved methyltransferase motifs. For instance, the PWWP motif of gene 1 and gene 2 are 88% and 84% similar at the amino acid and nucleotide levels, respectively, but considerably less similar to the other dnmt3 sequences (*e.g*. dnmt3-2 vs gene 4, accession #196918, has 64% and no significant similarity at the amino acid and nucleotide levels, respectively) (BLAST, NCBI)).

Recent additions to the sequence databases included two zebrafish sequences that appear to correspond to the same two genes and were named dnmt3 and dnmt5 respectively (GenBank numbers AB196914, AB196916). Our sequencing data corroborate the sequences submitted to the databases except for a few minor variations in regions with triplet repeats which may be an artefact of polymerase slippage in cloning or represent real triplet repeat differences that exist in the gene.

The high homology between dnmt3-1 and dnmt3-2 relative to other zebrafish dnmt3's, as well as their close proximity, suggests that these genes represent a duplication event. Postlethwait *et al*. [15] provides support for a model where two polyploidization events occurred in a common ancestor of zebrafish and mammals. However, there are often additional multigene members in zebrafish. Postlethwait *et al*. [15] argues that either chromosome duplication or another tetraploidization event in the zebrafish lineage is the most likely mechanisms by which these additional members arose. The tight clustering seen here, however, suggests that, at least in this instance, tandem gene duplication has occurred.

**Figure 3:**
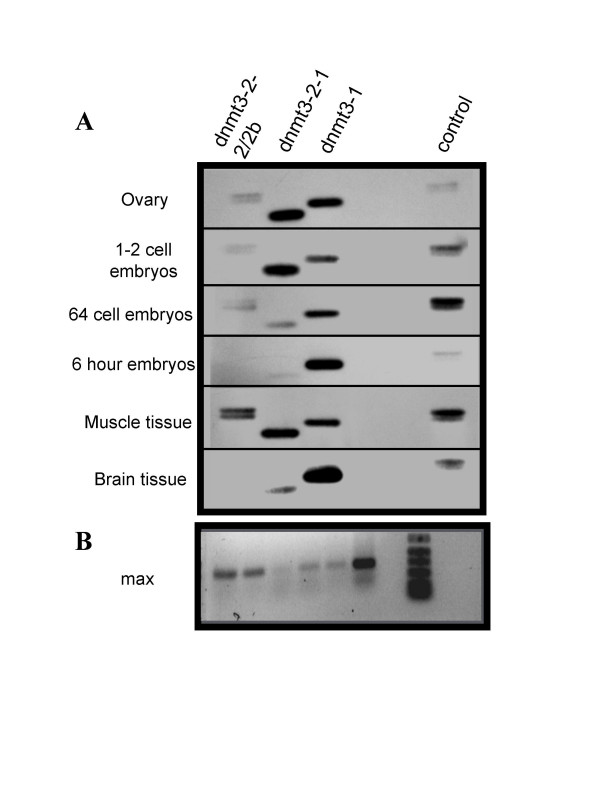
**dnmt3 isoforms**. A) RT-PCR followed by agarose gel electrophoresis and hybridization with a biotin-labelled probe. Stages/tissues used are labelled on the left side of figure. The first lane in all cases contains a doublet representing the two splice variants of dnmt3-2 differing in size by 78 bp. The second lane shows the alternate translational start site variant of dnmt3-2. The third lane is the product of the primers specific for gene 1 and the last lane is a control reaction loaded on each gel to allow comparisons between gels. The amount of reaction loaded was not the same in all lanes but was varied to produce more equivalent band intensities for more accurate quantification. Controls lacking reverse transcriptions produced no amplification products (not shown). B) RT-PCR of a constitutively expressed gene, max, for each RNA used serving as an internal standard for quantification. Lanes 1–6 show the max amplicons generated from the samples used in panel (A), ovaries through to brain. Lane 7 contains size markers.

**Figure 4:**
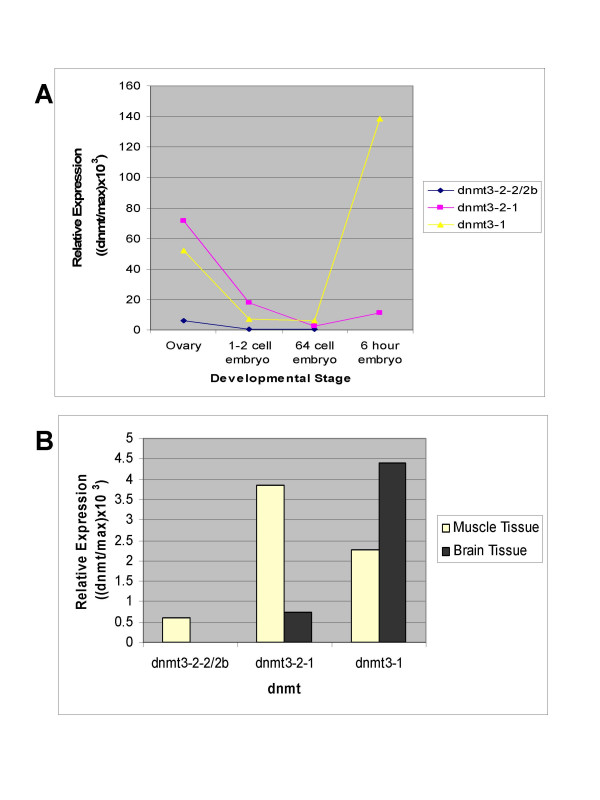
**Expression Summary**. A) Graph showing data from figure 3 developmental stages corrected for differences in amounts loaded, and normalized to max to correct for concentration differences as well as the control for exposure differences (see methods). B) Graph showing data from figure 3 somatic tissues corrected as above and demonstrating the relative expression levels of the three transcripts in those tissues.

**Figure 5:**
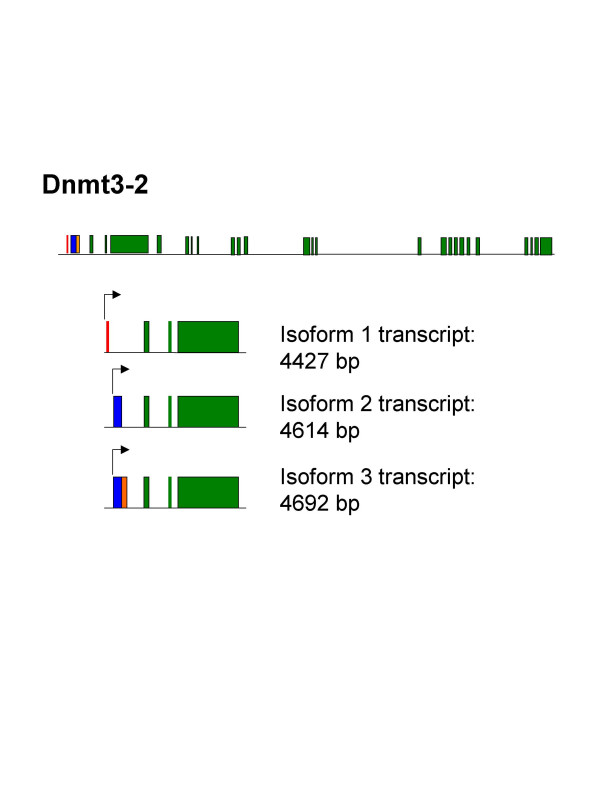
**The various transcripts produced from Gene 2**. The genomic structure is presented on the top line, with the 5' region of interest magnified below illustrating the various alternative splicing products. Transcript 1 (line 2) differs from the others by an alternate transcriptional start site and a missing exon 2. Transcripts two (line 3) and three (line 4) lack the first exon and are alternatively spliced in the second exon (the one missing from transcript 1). All splice variants occur upstream of the AUG start site.

The most interesting aspect of our analyses is that at least one of the genes, dnmt3-2, includes at least two start sites and a number of splice variants. These were initially identified in cDNA libraries generated from 1–2 cell embryos and RACE-PCR and were later confirmed by RT-PCR in a number of early embryonic zebrafish stages as well as somatic tissues (figure 3). This demonstrates that they are all expressed at least to the level of RNA. Densitometric analysis revealed that the transcript levels are not equivalent and that the relative levels of the different genes and isoforms fluctuate independently between the stages examined (Figure 4). All genes and variants examined are expressed in early embryonic stages, though dnmt-3-2-1 appears to be the most significant prior to zygotic gene activation (zygotic gene activation occurs at ~3 hours). All transcripts demonstrated declining levels leading up to this event, suggesting maternal supply turnover. Following zygotic gene activation however, there appears to be a marked shift towards dnmt3-1 being the most highly expressed. Additionally, there appears to be tissue dependent differences in expression levels (Figure 5). These differences in expression profiles for the different gene products and isoforms suggest that they are regulated independently and each may be playing distinct and separate roles during the development of the zebrafish.

The shortest of these variants, dnmt3-2-1, corresponds to the dnmt5 sequence in the database. The two novel variants reported here differ in size from that sequence by 187 (dnmt3-2-2) and 265 (dnmt3-2-2b) base pairs. These variants are actually associated with the gene having the most restricted promoter region. A schematic of the three products is shown in figure 5.

There are several interesting aspects of these dnmt3-2 variants. To begin with, although the splicing difference between variant dnmt3-2-2 and dnmt3-2-2b appears to involve the same 3' splice junction it has a different 5' splice junction, meaning that one of those splice sites is located within the exon of the other variant. However, both of the junctions still abide by the GT/AG rule for splice junctions.

The second interesting aspect of these splice variants is that all of them are 5' to the initiator AUG. Therefore, none of them actually affect the amino acid sequence. This suggests that either the splicing differences are trivial or they play a regulatory role in the translation or localization or some other aspect of the various splice variants. The latter possibility is a more reasonable assumption since, parsimoniously, it seems unreasonable to assume that this RNA would be alternatively spliced in a variety of ways for no biologically relevant reason. This situation is not unique to zebrafish dnmt3 genes. Similar splice variants in the 5'untranslated region have also been reported for human DNMT3s [13].

## Conclusion

We have isolated and analysed several of the dnmt3 genes from the zebrafish. In this report we have focused on two of the genes that are located in close proximity in a single linkage group and we find that the two genes are considerably more similar to each other than they are to the other zebrafish dnmt3 genes. This suggests that they arose as a result of a relatively recent gene duplication event. We have also found evidence for the existence of several different splice variants and alternative splice sites associated with one of the two genes, reminiscent of what is seen with the human DNMT3s. Expression analyses of these genes and variants demonstrate that are dynamic during development with distinct patterns that suggest they are independently controlled and, possibly, have different functions in development.

## Methods

Total RNA was isolated from ovarian tissue using the phenol/chloroform method of Chomczynski and Sacchi [16]. Ovarian Poly A^+ ^RNA (FastTrack 2.0 kit, Invitrogen Inc. Carlsbad, CA) was used for first strand cDNA synthesis using the BD SMART™ RACE cDNA Amplification Kit (Clontech, Palo Alto, CA). Using the zebrafish dnmt3 EST (GenBank number AF135438), a gene specific primer, GSP1 (see Table 1 for primer sequences), was designed to amplify, along with the universal primer, the relevant cDNA using PCR conditions as described by the manufacturer. Based on a resulting sequence that corresponded to four different regions of the genomic map (The Sanger Institute Welcome Trust zebrafish sequencing project), a series of gene specific primers were designed for the various genes.

**Table 1:** Primers used

Primer name	Sequence
GSP1	5'- GACAGGACCCTGAATGGACGTCGCT
GSP2	5'- GAGAGAGCACTGAGATGTCAG
GSP3	5'- CCAGAAATCTGTTGGAGACATTACACC
GSP4	5'-AAGGCAGTATGGAGTCTGTCTGCA
GSP5	5'-CAGTCATGGCAATGTCTTTCC
GSP6	5'-ATGTATGTCCTGTGAGGAGGAAC

PCR products were fractionated on 0.8% agarose gels, visualized with ethidium bromide, excised from the gel and cloned into pCR 2.1 vectors (TOPO TA cloning kit, Invitrogen Inc. Carlsbad, CA). The cloned products were then purified (Wizard^®^Plus Minipreps, Promega Inc. Madison, WI), and sequenced (Cortec DNA Service Laboratories Inc., Kingston, ON).

Reverse Transcription-Polymerase Chain Reaction (RT-PCR) was used to determine the relative expression levels of gene 1, gene 2, and its variants in tissues. Total RNA from zebrafish ovarian tissue, 1–2 cell embryos, 64 cell embryos, 6 hour embryos, muscle tissue, and brain tissue was isolated as described above, and the integrity checked by ethidium bromide staining. The RNA was then reverse transcribed using M-MLV Reverse Transcriptase (Invitrogen Inc. Carlsbad, CA) using primers specific for the various genes and variants. GSP2 was used for first strand cDNA synthesis of Gene 1 in conjunction with GSP3, generating a predicted amplicon of 521 bp. Primer GSP4 designed to anneal to all three gene 2 variants was used with GSP5 for dnmt3-2-1 to produce a 420 bp amplicon and with GSP6 to produce two amplicons of 597 bp and 675 bp from gene 2 variants dnmt3-2-2, and dnmt3-2b. In addition, RT-PCR was conducted to generate a 440 bp amplicon with GSP7 and GSP8, primers specific for a constitutively expressed gene, max [17]. PCR reactions were set up as described by the manufacturer, except that 2 ul of cDNA template were used for each reaction. PCR conditions were designed to ensure that all amplifications were within the logarithmic phase. Those conditions were; 94°C for 1 min, 25 cycles of 94°C 30 sec, 59°C for 30 sec, 72°C for 1 min, and a 72°C for1 min final extension for all primer sets except max which was only amplified for 14 cycles. Controls lacking RT were run for each RNA sample.

RT-PCR products were separated on a 1.5 % agarose gel, transferred to nylon membrane (Roche, Indianapolis, IN) and visualized by hybridization with a biotin labeled sequence designed to hybridize to gene 1, gene 2, and the variants (North2South Biotin labeling kit, Pierce Biotechnology Inc. Rockford, Il). Densitometric analysis of autoradiographs was performed to determine the relative expression levels of the genes and their isoforms at the above mentioned zebrafish developmental stages and tissues. Samples could be compared on different blots by using a control sample present on each autoradiograph, and samples were calibrated using the endogenous control max.

Zebrafish care and feeding was performed essentially as described by Westerfield [18]. All experimentation was done with the approval of the Canadian Council on Animal Care.

## Authors' contributions

THLS completed the isolation of several of the zebrafish dnmt3 genes and performed the expression analysis identifying the isoforms. CCD first identified that numerous dnmt3 genes exist and isolated a portion of two of them. AAM initiated this work and isolated the first dnmt3 sequence from the fish. RAM conceived the study and isolated portions of a number of the dnmts. All had intellectual and design input into portions of the experimentation. All authors read and approved the manuscript.
